# Socio-ecological influences of leisure-time physical activity among Nepalese adults: a qualitative study

**DOI:** 10.1186/s12889-021-11484-3

**Published:** 2021-07-22

**Authors:** Susan Paudel, Alice J. Owen, Ben J. Smith

**Affiliations:** 1 School of Public Health and Preventive Medicine, Monash University, 553 St Kilda Road, Melbourne, Victoria 3004 Australia; 2 Sydney School of Public Health, The University of Sydney, Sydney, Australia

**Keywords:** Leisure, Physical activity, Focus groups, Thematic analysis, Socio-ecological model

## Abstract

**Background:**

With economic and social changes, participation in occupational and transport-related physical activity is declining among Nepalese adults, highlighting the growing importance of leisure-time physical activity. However, limited information is available to guide public health policies and interventions to promote leisure-time physical activity in Nepal. This study aimed to qualitatively explore the socioecological influences of participation in leisure-time physical activity among Nepalese adults aged 40 years and above.

**Methods:**

A total of 51 adults (30 females and 21 males) participated in one of the nine focus groups conducted in Kathmandu, Nepal. A semi-structured guide based on the social-ecological model of physical activity was used to facilitate these focus groups. Data were analysed using a reflexive thematic analysis approach in NVivo 12.

**Results:**

Participation in leisure-time physical activity was minimal and leisure time was mostly spent resting, socialising, or engaging in sedentary activities such as watching television. Walking was the most common form of leisure-time physical activity, and men reported being more active than women. Individual-level barriers included lack of knowledge, lack of skill, lack of motivation, considering oneself as sufficiently active and engagement in sedentary screen activities. Family and household responsibilities, lack of support and fear of being judged constituted the interpersonal barriers while environmental barriers included an absence of a supportive social norm, lack of open spaces, weather conditions and perceived lack of safety. Health benefits, prioritising physical activity, social support, provision of group-based activities and age-appropriate public exercise facilities were identified as major facilitators.

**Conclusion:**

Critical issues that need to be addressed to increase leisure-time physical activity among Nepalese adults include traditional gender roles, family and social support, and social norms. Modifications of the built environment, such as public exercise facilities, offer further opportunities and will require coordination beyond the health sector.

**Supplementary Information:**

The online version contains supplementary material available at 10.1186/s12889-021-11484-3.

## Background

Leisure-time physical activity (LTPA) includes diverse activities carried out as part of exercise, recreation or sports, usually performed in short bouts [1, 2]. Previous studies have documented the health benefits of LTPA, such as the reduced risk of all-cause and cardiovascular disease (CVD)-related mortality [3, 4], risk of incident CVD [3], type 2 diabetes mellitus (T2DM) [5] and several types of cancer [6, 7]. Furthermore, regular LTPA can reduce the risk of dementia [8] and promote life satisfaction and psychological well-being in adults [9, 10]. However, in many countries, engagement in LTPA is relatively more common among younger age groups and decreases with advancing age [11]. Furthermore, women are less likely to participate in regular LTPA than men [11].

In Nepal, the overall prevalence of physical inactivity is around 7.4% (6.6% in women and 8.2% in men) [12]. Consistent with other South Asian countries [13], occupational and transport-related activities are the most prevalent forms of physical activity (PA) while engagement in LTPA is minimal [12]. Overall, the percentage contribution of LTPA to total PA was 1% among males and 0.3% among females aged 40 years and older [14]. The retired or unemployed, those with higher education and those from affluent households were most likely to engage in LTPA [14].

While there is a large body of evidence about the role played by PA in the prevention and management of chronic diseases [5, 15–17], a recent study reported that PA only had an indirect relationship with T2DM among Nepalese adults, which was mediated by waist circumference [18]. It was postulated that this unexpected finding was possibly due to the low participation levels in LTPA among Nepalese adults [18]. Global research has recognised that the physical and mental health benefits of LTPA are more than those offered by other domains of PA, such as work, transport, or household PA [2, 19–22]. This highlights the need to examine and understand LTPA even if there is markedly higher engagement in other domains of PA, such as in Nepal.

In addition to this, participation in occupational and transport-related activities, which are the dominant forms of PA among Nepalese adults, are likely to decrease in the future because of the expansion of road networks, increasing preferences and access to motorised transport (both public and private), decreasing agrarian land and mechanisation of household and farm-based activities [23]. Promotion of LTPA is required to prevent the likely increase in physical inactivity, which is an undesirable consequence of the declining levels of engagement in other domains of occupational and transport-related PA [23]. This will require a better understanding of the factors hindering or facilitating adults’ participation in LTPA to provide an evidence-base for PA policy and programs. The socio-ecological model recognises the influences upon individual behaviour operating at multiple levels [24]. Bauman and colleagues [25] have applied this model to identify the determinants of PA at the individual (such as psychological and biological factors), interpersonal (such as social support from family, friends and workplace), environmental (such as social, built and natural environment), regional or national policy (such as transport systems, urban planning, PA policy) and global levels (such as economic development and urbanisation). An understanding of all these levels of influence specific to the domains of PA is crucial to design and implement successful interventions. However, studies providing this information among Nepalese adults, with a focus on LTPA, are absent. Hence, this study aimed to qualitatively explore the socio-ecological barriers and facilitators of LTPA among middle-aged Nepalese adults.

## Methods

The Monash University Human Research Ethics Committee (#18575) and the ethical review board of Nepal Health Research Council (#2803) approved this qualitative study. We have reported the findings following the Consolidated Criteria for Reporting Qualitative Research (COREQ) checklist [26].

### Study design

A descriptive qualitative approach was used to explore the socioecological barriers and facilitators to LTPA among Nepalese adults. Nine focus groups were conducted in three purposively selected wards of Budhanilkantha Municipality. The use of a focus group design enabled discussion among the participants to compare and contrast their views and experiences, generating deeper insights into the influences upon LTPA [27, 28]. The study area is one of the 11 municipalities of Kathmandu district, Nepal’s capital, with 153,000 inhabitants across its 13 wards [29]. Located at around 30-min medium-paced drive from the capital centre, the municipality is rapidly urbanising and has witnessed a shift in agricultural activities in the last few years.

### Participants and recruitment

Participants were community-dwelling males and females aged 40 years or above. Middle-aged adults were selected for this study because of the declining levels of PA and higher risk of chronic diseases in this group [12]. Those with chronic diseases (cardio-metabolic conditions such as diabetes, hypertension and raised cholesterol) and those free from these conditions participated in separate focus groups. It was expected that participants with these conditions will have relatively higher PA knowledge and participation. Hence, they were included in separate focus groups to prevent the influence this may have on the other member’s responses. People who were unable to engage in PA on a routine basis and those unable to consent were excluded.

A purposive sampling technique was used to select participants for this study. Mother’s group members and female community health volunteers were the first contact point at the study site for participant recruitment while local male leaders helped in recruiting male participants in the study. These groups contacted potential participants to explore their willingness to participate in the study. If the potential participants showed interest, their contact information was then shared with the research team. A member of the research team then contacted potential participants to explain the study further, assess their eligibility, and discuss the focus group process, times, and venues. The focus groups were conducted between September–December 2019. Participant recruitment and data collection stopped when no new information emerged (data saturation) on preliminary data analysis carried out concurrently with data collection [30].

### Data collection

The focus group discussion guide (see supplementary file 1) was structured to explore the barriers and facilitators of participation in LTPA. The participants with chronic conditions were also asked to share their LTPA participation experiences pre- and post- their diagnosis. A translated version of the guideline in the Nepali language, pre-tested with a similar group of participants, was used during the focus groups. This discussion guide was developed based on a literature review and was primarily guided by the socio-ecological model of the determinants of PA [24, 25].

Out of the nine focus groups, four groups consisted of male participants, while the remaining five consisted of females. In the context of power differences between males and females and the gender norms in the Nepalese context, it was vital to have separate focus groups so that the female participants would feel safe and comfortable in sharing their perceptions and experiences. Each focus group consisted of 5–6 participants and lasted for around 30–45 min. Focus groups took place at a mother’s group meeting hall or a home setting easily accessible to participants. A member of the research team (SP), trained in qualitative research methods, moderated the discussions in the Nepali language, while a research assistant, native to one of the study communities, supported with notetaking. Probing questions were asked for clarification and to prompt further discussion and explanation until all conversation subsided.

At the onset, participants were informed about the study purpose, study methodology, risks, and benefits of participation, and written informed consent was obtained. This was then followed by collecting demographic information, such as full name, age, education, marital status, and main occupation, before the group discussion. After each focus group session, the moderator debriefed the participants’ key points to ensure the discussion had been correctly captured. All the sessions were audiotaped with permission. The participants were provided with a lunch pack as a remuneration for their time.

### Data analysis

Focus groups were transcribed verbatim using the audio recordings and translated into English by a member of the research team (SP). All personal identifiers were removed, and codes were assigned before analysis. The translated transcripts were analysed using a reflexive thematic analysis approach [31]. Reflexive thematic analysis “emphasises the importance of the researcher’s subjectivity as an analytic resource, and their reflexive engagement with theory, data and interpretation” [32]. It is a six-phased process and includes familiarisation with the data, coding, generating initial themes, reviewing themes, defining and naming themes and writing up [33]. While the shared language and cultural background of the lead researcher (SP) and study participants was highly valuable, it was recognised that caution needed to be taken during analysis given the researcher’s differing life experience as a younger, urban-dwelling and university educated person. For this reason, the debriefing of participants after each focus group played an important role, to check that the issues discussed had been properly recorded, as did the adoption of a reflexive, iterative process during coding.

The coding (undertaken by SP) commenced using deductive methods to identify codes corresponding to the individual, interpersonal and environmental influences upon LTPA as outlined in the socio-ecological model [25, 34]. The codes were then discussed within the research team and refined. This included merging specific codes to generate general higher-level codes. Relevant themes aligned with the socio-ecological model were generated and approved by the team. An inductive process was used to identify any additional barriers and facilitators of participation in LTPA. Data was managed using NVivo 12 plus (QSR International, Melbourne, Australia).

## Results

A total of 30 female and 21 male participants participated in nine focus groups. Among these, 14 female and 10 male participants self-reported suffering from at least one cardiometabolic conditions (e.g., diabetes, hypertension or raised cholesterol). The participants’ ages ranged from 40 to 60 years, with the majority being 50–59 years of age. Male participants were mostly educated up to lower-secondary or secondary level and were either retired or unemployed. In comparison, most female participants had primary or a lower level of education and were homemakers. Most of the participants were of advantaged ethnic groups, such as Brahmin, Chhetri or Newar. These ethnicities were classified as belonging to advantaged groups because of their relatively higher education, occupation, or economic status.

### Engagement in LTPA

Participants reported minimal engagement in LTPA. They did not perceive that leisure time was for engaging in some form of PA but instead viewed it as a time to rest, rejuvenate and complete errands. Among those who engaged in LTPA, walking was the most frequently reported activity. A male participant shared, *‘On most of the days, I walk between 1-2 hours in the morning. This is the main form of exercise for me’.* Most participants preferred walking in the mornings rather than afternoons and evenings. A female participant stated, ‘*I feel walking in the early morning gives us more benefits and makes us feel better than walks in the afternoon or day time….the fresh air is the added benefit during morning walks’.* Participants suggested that they more frequently encountered those with health conditions, retirees and relatively older people (above 50 years) in these morning walks. This was mostly related to a greater availability of time for those who were not working and a motivation among those with chronic diseases to avoid a worse prognosis.

Dancing and yoga were other forms of LTPA mentioned by a few women. Reference was made to a mother’s group that organised some cultural programs every fortnight, which provided an opportunity to sing and dance. Some participants, particularly those with chronic conditions, reported engaging in light exercises at home, but not regularly. A hypertensive male participant stated, *‘I walk for 45 minutes in the morning and then exercise [yoga, stretching, and stationary running] after returning home for around 15-20 minutes’.* Another female participant suffering from diabetes added, *‘After diagnosis, I am compelled to do some exercises and go for walks.’*

There were distinct differences in how males and females reported spending their leisure time. For women, afternoons (post-lunch) were slightly relaxed and were utilised by taking naps, watching television, using the internet, or doing other household chores such as preparing vegetables for dinner or cleaning the house. A female participant who was engaged in formal employment stated, *‘I never go on random walks even if I am free. I rather engage in detail cleaning of the house and activities related to food processing or preparation’.* Some of the participants also mentioned chatting with friends/neighbours as their leisure time activity.

Conversely, most male participants utilised their leisure time outside of the home. A male participant said, *‘We usually gather at shops, discuss social and political issues and sometimes play games (cards)’.* Gathering at local tea shops, chatting with friends, and playing games such as cards or carom (or watching others play) were the common leisure-time activities among males. Another male added, *‘Afternoons are usually relaxed. I will be resting at home or will go to nearby shops. Other people also gather there. We spent time having tea or chatting with friends. Some people also play cards”.*

### Barriers to LTPA

Individual-level barriers to LTPA that were discussed included lack of knowledge, lack of skill, lack of motivation, considering oneself as sufficiently active, and screen time. Family and household responsibilities, lack of support, and fear of being judged constituted the interpersonal barriers, while environmental barriers included an absence of a supportive social norm, lack of open spaces, parks and exercise facilities, weather, and lack of safety (see Table 1). Some of the barriers were consistent across the groups, while some disproportionately affected females, those with chronic diseases, and different age-groups. These have been further explained below.

**Table 1:** Barriers and facilitators of participation in LTPA among middle-aged adults in Nepal: an adaptation of the socio-ecological model

	Individual level	Interpersonal	Environmental
**Barriers**	• Lack of knowledge • Lack of skill • Lack of motivation • Considering oneself as sufficiently active • Screen time	• Family and household responsibilities • Lack of support • Fear of being judged	• Absence of supportive social norm • Lack of open spaces, parks and exercise facilities • Weather • Lack of safety
**Facilitators**	• Health benefits • Prioritising PA	• Social support	• Availability of group activities • Public exercise facilities

#### Individual-level barriers

##### Lack of knowledge

Participants were relatively active in household and transport-related physical activities and demonstrated a lack of awareness regarding the benefits of engaging in LTPA. Lack of knowledge was relatively more common among older participants. On the other hand, participants with chronic conditions seemed to be more aware about LTPA as exercise was a crucial component of their disease management plan and their treating physicians usually had explained this to them. A male participant commented, *‘People are not aware of its [LTPA] importance, and they do not realise the harm of missing it out. We feel lazy. We have not realised that engaging in exercise will give us extra benefits’.* There was also a lack of awareness regarding the types of age-appropriate activities that could be carried out indoors, in the limited space available. Another male from the non-diseased group added, *‘The main reason for not engaging in exercises is primarily because of the lack of knowledge about its importance, types of exercises that can be done in a limited space and lack of skill’.*

##### Lack of skill

Walking was the most common form of LTPA among the participants because it required neither any specific skill nor much planning or resources. A female explained, *‘Like for morning walks, we do not need to think a lot. We can just wake up and then walk. However, exercising needs space, skills, and planning, and we lack this’.* Both male and female participants expressed that the lack of skills, for yoga or other age-appropriate exercises requiring minimum space, limited their participation in LTPA. A male participant said, ‘*For yoga, we do not have the skills, and there is no one to train us. With increasing age, our walking ability will be limited. Then, we will need some skills to keep us moving inside the house, and yoga can be one of them’.*

##### Lack of motivation

Most of the male and female participants commented that they felt ‘lazy’ and were not motivated to engage in LTPA. This was mainly found to be an issue among those without chronic diseases. A male participant said, *‘Space is not only an issue. We can exercise or do yoga inside the home as well. However, we are not doing it because we are not motivated or get lazy’.* For those with chronic diseases, exercise (e.g., morning walks) were often prescribed as part of their disease management plan, and they shared that they felt compelled to engage in these activities. However, a diabetic female participant shared, *‘Laziness is also stopping us. We can manage time if we wake up slightly early in the morning but are too lazy. I think we are not motivated enough, which in turn is making us lazy’.* Some participants with chronic diseases expressed a lack of concern about the health risks of not exercising. A female participant who was suffering from hypertension and raised total cholesterol stated, *‘I sometimes feel, even if I die by not exercising, I do not care’.*

It was apparent that a lack of motivation was associated with a perceived lack of time to undertake LTPA. As one male participant stated, *‘For people like us who are employed in 9 to 5 jobs, it is difficult to find leisure time during weekdays. Mornings walks are not regular for me because sometimes I get lazy or occupied with some other works or am getting late for office’.*

##### Considering oneself as sufficiently active

Participants reported being relatively active in the household, occupational, and transport-related domains, and hence, they conveyed the viewpoint that it was not necessary to engage in LTPA. Female participants without chronic disease most often expressed this opinion, with one commenting: *‘I have not been able to go for the morning walks. However, as part of our household chores in the morning, our movement within the house works as morning walk’.* Another woman explained, *‘We think household activities provide with required exercise, so there is no need to engage in extra’.* There was also the viewpoint that, because of housework’s physical demands, leisure time needs to be used to rest. However, those diagnosed with chronic diseases were more conscious about engaging in LTPA, particularly for morning walks. A female participant suffering from diabetes said, *‘Previously, house works would be enough as we used to engage in a lot of labour-intensive activities. However, now I do not think it meets the requirement’.*

##### Screen time

Access to smartphones and the internet was reported to be displacing active forms of leisure among both male and female participants. Relatively younger and educated adults mostly spent their leisure time by engaging with their phones. Facebook and YouTube were the most common platforms. A male participant said, *‘If I get some leisure time, I usually spend it by watching television, using my phone or chatting with friends in the local shops’.* Females, who otherwise have a hectic household routine, were also increasingly being sedentary with increased access to smartphones and the internet. Another male added, *‘Previously, my wife used to be active throughout the day. These days, I see her busy on Facebook whenever she gets some free time’.*

#### Interpersonal barriers

##### Family and household responsibilities

Female participants reported that the household responsibilities of cooking, cleaning, and looking after the family created hindrances to LTPA. Morning walks were the most common form of LTPA; however, the morning hours were often hectic for females. A female participant shared, *‘We want to go for walks, but we also have to finish household chores. In most cases, we prioritise household tasks to morning walks’.* Another female stated, *‘I used to go for morning walks, but I stopped because of household chores’ burden’.* The afternoon breaks provided some rest from the hectic morning and evening routine. Women stated that they had to wake up at least an hour earlier if they wanted to manage time for these walks. Though feasible during summer, this was challenging during the winter. However, the older females in the house had relatively more leisure time if there were younger females in the house to carry out these responsibilities.

##### Lack of support

Lack of support from family members in sharing the burden of household work and the lack of partners to exercise or walk with hindered women’s participation in LTPA. Already tired upon returning from morning walks, women struggled to finish all the household chores with nil or minimal support from other family members. A female participant mentioned, *‘Upon returning, we cannot even rest for a while. We need to start completing all the unfinished errands. This then gets hectic and more tiring’.* Most of the participants reported a lack of motivation to exercise alone. A female participant suffering from hypertension said, *‘Every time I visit the doctor, exercise is recommended, but I am still not adhering to it. I do not know why. During the conversation, I feel motivated. I think I will start immediately. However, when I reach home, I do not feel like exercising alone’.*

##### Fear of being judged

Female participants shared that they were afraid of being judged by family or community members, limiting their engagement in LTPA. A female participant said, *‘Whenever I am dancing, I lock the door. I cannot keep the door open because I feel shy. On top of that, my kids will not like the songs I will be dancing on’.* In many instances, they reported feeling shy and embarrassed, even though their family members had not complained or expressed any judgement. Another female living with a cardio-metabolic condition explained, *‘I sometimes dance in religious songs when my sons are still sleeping. I cannot do that when my sons are awake. Though they will not tell anything, I feel shy’.* They were concerned that the noise of the music and dancing steps would disturb other family members. Some participants shared that this was likely to happen when other family members were not aware of the types and benefits of LTPA. A female participant stated, ‘*Some are not aware of the benefits of exercise, and they might not like when other family members are exercising’.* Some participants also reported concern about being judged by community members: *‘If we [women] want to do something new, people will start gossiping and demotivating us’.*

#### Environmental barriers

The environmental barriers included the factors related to the social environment (such as social norms), built environment (such as parks and recreational facilities) and natural environment (such as weather). These barriers have been further explained below:

##### Absence of supportive social norm

Besides walking, engagement in LTPA was reported to be uncommon among middle-aged and older adults. Instead, not engaging in LTPA was considered the prevailing norm. A male participant said, *‘We are not engaging also because it is not a common practice. We do not see people around us exercising or doing other physical activities in their leisure time’.* Participants commented that if there were examples around them, it would be a learning opportunity. Another male stated ‘*We rarely see people of our age engaging in such activities around us. If there were, we could learn from them and get motivated’.* Instead, engaging in sedentary activities such as watching television, chatting, gathering at local shops, and playing cards were everyday leisure time activities. Considering walks as a morning event also limited recreational walking during other times of the day. A female participant shared, *‘Going for a walk is mostly considered a morning event when we are usually busy in our household activities. We are usually free during the afternoons and going for a walk at this hour is not common’.*

##### Lack of open spaces, parks, and exercise facilities

Participants reported a dramatic decline in open spaces in recent years. Many participants reported no open spaces or parks to engage in LTPA in their neighbourhood, which had limited LTPA for all age groups. A male participant stated, *‘Even if we want to, there is no open space around. When we were children, we used to engage in many outdoor sports. We used to be mostly outdoors, but kids these days are mostly indoors. There is no open space around for outdoor activities’.* Another male further added, *‘The outdoors games that we used to play are almost extinct in urban areas because of the lack of open space’.* Private gyms mostly targeted youths and were not affordable to everyone, while there were no public or community gyms. In line with this, a male participant shared, ‘*Age-friendly gym and fitness activities are not available. Gyms are not near to our home, and they are also not affordable to everyone’.* Females particularly showed interest in Yoga and a female participant shared, *‘There was a yoga camp nearby the place I was living before. I used to attend the morning sessions regularly, but there is no such option nearby here’.*

##### Weather

Extreme weather conditions further challenged participation in LTPA. Females reported that waking up early for morning walks was difficult during the colder and rainy months. Road conditions were reported to deteriorate during the monsoon season, making walking arduous. A male participant said, *‘On rainy days, the walking trails and even the roads get quite muddy, making it difficult to walk around’.* The monsoon season was particularly challenging in areas where there were unsealed roads or potholes in the road, and people had to use alternative routes. A female participant added, *‘Though the level of pollution decreases during the rainy season, walking is not easy. Hence, we usually walk different routes in dry and wet seasons such as we walk around the paddy fields/walking trails in the dry season but walk in the roads during the wet season’.*

##### Lack of safety

Participants reported feeling unsafe from stray animals, theft, or strangers in poorly lit neighbourhoods. This significantly interfered with walking during the darker winter mornings. A female participant stated, *‘We do not feel safe in areas with poor street lighting. We are scared of stray dogs. Sometimes we also encounter drunk men. Walking alone does not always feel safe’.* Another man said, *‘There is also a risk of stray dogs. It was quite severe earlier, but now, the municipality is working on its management’.* Insufficient street lighting not only made participants feel unsafe but also made walking difficult. A male participant mentioned, *‘There are potholes in the road which we cannot see in the dark. In the rainy season, they will be filled with muddy water, and we cannot even ascertain their depth’.*

### Facilitators

Focus group participants mostly focused on the barriers to LTPA and generally considered removing those barriers would create a conducive environment for LTPA participation. There were, however, several factors such as health benefits, prioritisation of PA, social support, provision of group-based activities, and age-appropriate public exercise facilities, which were identified as motivators and enablers of LTPA. These facilitators are further explained below and summarised in Table 1.

#### Individual-level facilitators

##### Health benefits

Awareness and experiences of health benefits from LTPA were reported to motivate participants. It was mainly those with chronic conditions who were conscious about attaining the prescribed amount of PA, yet a female participant expressed the view, *‘Those who are not yet diseased need to be more active as those who are diseased are already diseased now’.* Further, some participants were aware that since their PA in the household and transport-related domains had decreased, they needed to engage in alternative PA forms. A male participant commented, *‘I understand that our activties have drastically decreased compared to the past, and the only way we can close this difference is by engaging in leisure time activities. However, we are not doing it. We are becoming increasingly sedentary’.*

##### Prioritising PA

While some participants perceived that they lacked time for engaging in LTPA, others considered it was more related to prioritisation than insufficient time. For instance, it was noted by female participants that if they wake up an hour earlier in the morning, they would have ample time to go for morning walks and to finish their morning household tasks. Females generally prioritised household responsibilities over doing something for themselves; whereas a few women thought they could manage both if they were motivated enough. A female participant mentioned, ‘*Neither we have fields, kitchen garden nor have to fetch water. Main morning chores are cooking and sweeping the house. We can do these works simultaneously. We can manage time if we are motivated to going for a walk in the morning’.* Some male participants perceived that a daily schedule would help them undertake regular PA. A male participant said, ‘*If there were a schedule that would explicitly mention our everyday activities from when we wake up to when we exercise, we probably would be exercising’.*

#### Interpersonal facilitators

##### Social support

Females reported that family members’ support, in either sharing household responsibilities, moral support or exercising together, was instrumental for remaining active. Male focus group participants also observed that women with more support were likely to walk more regularly. Females perceived that if other family members were engaging in LTPA, it was more likely that they would feel more supported and motivated. A female participant stated, *‘If some other members of the family were exercising, we would be motivated’.* Both male and female participants stated the importance of peer support. Having a “push” from friends helped them to be active. A male participant said*, ‘If a friend comes to us and asks us to join them, we will readily agree. However, very few walk during leisure’.* Another female added, *‘If we can find some friends, walking in the afternoon when we are usually free would be a good idea’.* Some female participants valued peer support for safety reasons: *‘For short distance and main roads, we do not need friends, but if we are going a bit far and side-roads in quiet neighbourhoods, we usually walk in small groups’.*

#### Environmental facilitators

##### Availability of group activities

Both male and female participants reported group-based activities and the role modelling they offered as facilitators. A male participant stated, *‘There is a park around 30 min drive from here. A trainer conducts structured stretching activities in the morning. People gather and exercise for around 30 min to an hour. If we also had such a thing, we might also attend’.* A female participant added, *‘I sometimes go to a camp in [a nearby suburb] where we dance for an hour. It runs the whole day. If we go in the morning, we can walk till there, which also acts a morning walk for me’.* Participants felt such group activities provided opportunities for social interaction, peer role modelling and helped them to stay motivated. Another female participant shared, *‘If there were structured activities such as yoga camps or a dedicated place and group-based activities, we would feel compelled to do. If all our friends were coming, we would also feel motivated or join them even if not motivated’.* Such activities were considered motivational and an opportunity to learn skills for age-appropriate PA. A male participant stated, *‘Knowledge and skills of exercises that can be done in a limited space will be useful as getting open space outside for engaging in sports is not always feasible’.*

##### Public exercise facilities

Male participants reported that public exercise facilities would provide both a motivator and an environmental enabler for LTPA. They were excited about such facilities installed in a nearby municipality. A male participant shared, *‘Recently in [nearby municipality], the ward offices have installed public gym equipment in some areas. We could exercise if we had that facility here. If we had that type of facility, we could have gone to those places instead of gathering at the shops and engaging in random chats’.* Another male participant who was also aware of such facilities being installed in another area added, *‘If we had it here, at least we could have gathered there and spent time at those places. We would have been motivated to be active. If we had such public spaces and facilities, we could play games as well’.* Highlighting the need for free public facilities in the neighbourhood, another male participant commented, *‘We cannot and will not be interested in paying for such services. Therefore, it should be free and easily accessible around our surroundings’.*

## Discussion

This is the first in-depth exploration of LTPA in Nepal, a country experiencing dramatic social and demographic change and increasing chronic diseases. The increasing levels of physical inactivity and declining participation in occupational and transport-related physical activities in Nepal mean that investigating the factors affecting participation in LTPA is essential and well timed. As proposed by the socio-ecological model, we observed interactions between the factors operating at different levels, which are explained below.

Among study participants, leisure time was perceived as a period for rest and relaxation, mostly utilised to socialize and engage in sedentary activities such as TV viewing, playing cards, or chatting. Participation in LTPA was minimal, and gender variations were evident, with men more likely to engage in LTPA. These findings support previous quantitative studies from Nepal, including the most recent World Health Organization Stepwise Approach to Surveillance (STEPS) survey [12, 14, 23, 35, 36]. Gender differences were also reported for walking, with men walking more regularly and allocating more time. Another cross-sectional study among Bangladeshi adults has also reported that women spend less time walking than men [37]. Those diagnosed with chronic conditions were more aware of engaging in LTPA besides regular household and occupational activities, consistent with a qualitative study from Sri Lanka [38].

Walking was the most common form of LTPA, with some engaging in light exercises, dancing, or yoga. A previous systematic review has reported that among Southeast Asian adults, participation in physical activities such as walking, running, cycling, and yoga is more popular than team sports [39], and our results are in congruence with this. Studies from India [40], Sri Lanka [38], and Bangladesh [41] have also reported walking as the most feasible and most common form of PA among adults. Our study participants mostly perceived walking as an early morning activity, although this was not always feasible for those who had hectic morning routines, especially women. Promoting walking can be a viable public health strategy to promote LTPA among Nepalese adults because of its popularity, benefits, and lower associated costs.

Considering oneself as sufficiently active, coupled with a lack of knowledge about the benefits of LTPA, affected participant’s interest and engagement in physical activities during leisure. Even those who were willing reported lacking the skills of exercises appropriate for their age and circumstances, including indoor exercises requiring minimum space. There was a recognition that structured group-based activities and training camps could provide participants with an opportunity to learn skills and be motivated through peer role modelling. It was notable that most study participants, even those with chronic conditions, reported that they felt lazy and lacked the motivation to initiate and sustain PA on their own. This finding agrees with a previous qualitative study among Indian women that reported that a lack of motivation and interest hindered participation in non-domestic forms of PA while observation of other walkers and training opportunities provided motivation [40]. Hence, future interventions might beneficially include group-based activities to provide an opportunity for peer role modelling and facilitate sustained behaviour change.

For women, the burden of household responsibilities was a key barrier, consistent with the findings of a previous qualitative study from India [40], a cross-sectional survey from Bangladesh [37], and an international review [42]. This mostly reflects traditional gender roles that allocate household work to women and teach women to prioritise family members over themselves. Participants in this study reported that a hectic household routine either left them with no time to engage in LTPA or in a state of exhaustion that deterred them from PA during their limited leisure time. Previous systematic reviews have also reported that household and family responsibilities left women with no time for PA and that taking time out from responsibilities for personal well-being (including participating in LTPA) was perceived as a selfish act by South Asian women [13, 43]. Women in formal employment further struggled to balance family and work responsibilities, leaving them with little time and energy for LTPA. Lack of time has been cited as a barrier for LTPA participation by women across many studies [37, 40]. On the other hand, support from family members in the form of assistance with household responsibilities and encouragement and/or motivation was recognised by women as a facilitator of LTPA. This highlights that knowledge and willingness among women may not alone be sufficient to initiate and sustain PA. Hence, an awareness about multiple hindering and facilitating factors operating within a family environment will be crucial to effectively plan and implement such interventions. Traditional gender roles and the availability of support and encouragement from family members have been found here to be particularly important issues for attention. Co-designing interventions with community members would be valuable for identifying relevant and acceptable strategies to address these complex social influences upon LTPA.

A significant insight to emerge from the focus group discussions was the apparent lack of a social norm for LTPA in Nepal. Participants, particularly women, felt uncomfortable and feared being judged by community members when exercising in public, and even by family members when exercising indoors. LTPA was mostly associated with youth, and seeing a middle-aged adult engaging in LTPA in public (besides walking) was considered a rare sight. Instead, engaging in sedentary activities or resting was a regular and expected activity during leisure. A previous cross-sectional study from Bangladesh has also reported that engaging in sports, exercises, or outdoor PA was not a social norm for adult women and recommended developing home-based exercise programs requiring minimum space and equipment [37]. Lack of social support, especially for women, has been consistently reported as a barrier for LTPA in studies from Asia [44, 45] and high-income countries [46]. Future interventions might consider raising awareness about the importance of LTPA and involving community members in planning and implementing these interventions to normalise engagement in various forms of LTPA.

Besides these individual, interpersonal, and social environment related barriers, built environmental factors such as the unavailability of parks, exercise facilities, and open spaces further limited LTPA participation, mostly for men. This finding concurs with results from a previous review among South Asian women [43] and quantitative studies from Bangladesh [37], Oman [44], and Hong Kong [47]. Poor street lighting and weather conditions created further hurdles. Stray dogs deterred women from morning walks, a barrier also cited in another study from India [40]. By contrast, a study among Bangladeshi women found that they preferred walking in the morning to avoid traffic and being seen by other people [41]. Study participants appreciated the recently installed public gym equipment in the nearby municipality and considered such provisions would provide a valuable motivator for LTPA. This would also help contribute towards normalising engagement in LTPA in public by people in various age groups. Continued structural changes of this type will require a multi-sectoral approach that engages policymakers outside of the health sector, such as urban planning and local government.

The study results highlight the interaction between various factors at the individual, interpersonal and environmental levels, as outlined in the socio-ecological model, that shape adults’ participation in LTPA and reinforce the need for multi-tiered and multi-level interventions to bring sustained behaviour change [24, 25]. Mainly, interactions were observed between factors such as lack of knowledge (individual level), lack of support and fear of being judged (interpersonal level), and absence of social norms (environmental level). Among females, considering oneself as sufficiently active at the individual level interacted with family and household activities at the interpersonal level. Females were overburdened with household and family responsibilities which were their main, and in most cases, only forms of PA. This hectic household routine made them exhausted, and they considered that, since they were already meeting the required levels of PA, there was no need to engage in non-domestic forms of PA.

### Strengths and limitations of the study

Most of the previous PA studies from Nepal are limited to examining total PA, while qualitative studies on LTPA are absent. In this context, the study findings will be valuable to sensitise policy makers on the growing importance of LTPA for Nepalese adults and guide public health policies and interventions. The use of qualitative data collection methods has helped to gain in-depth insights on the perceived barriers and facilitators, while the use of the gender and disease-specific focus groups provided an opportunity to explore barriers and facilitators of LTPA in important population sub-groups.

A limitation of this study is that participants were recruited from a rapidly urbanising area near the country’s capital. Further, most belonged to advantaged ethnic groups and these factors may have limited the range of insights concerning LTPA in Nepal that were obtained. A scheduling clash, lack of interest, and lack of time among male participants resulted in fewer study numbers than females. Besides, since the focus groups were translated and analysed in the English language, participants did not have the opportunity to validate the themes. However, at the end of every focus group, the researcher presented a verbal summary of the discussions to ensure everything was correctly captured. The study findings might not be generalisable to other age-groups as this study was only limited middle aged-adults. Furthermore, exploration of the broader policy and global factors outlined in the socio-ecological model was beyond the scope of this study.

## Conclusion

Participation in LTPA was minimal among middle-aged adults in Nepal, with those diagnosed with a chronic condition expressing the highest interest and engagement in this form of PA. The absence of supportive social norms, the burden of household responsibilities (particularly for women), lack of skill, insufficient time, and an unfavourable built environment were the key barriers that hindered participation in LTPA. Promoting LTPA participation among Nepalese adults will require a multi-tiered and multi-sectoral approach to address a range of individual, interpersonal, and environmental barriers. Future interventions need to address gender norms to ensure women get more support and time for engaging in LTPA. The development of public recreational facilities and group-based PA are promising opportunities for promoting LTPA in this context. Future studies exploring the broader determinants identified in the socio-ecological model such as regional or national policies related to transport and urban planning, the effects of commercial interests, and global influences upon economic development and urbanisation will provide further insights concerning factors that should be considered in the development of PA policies and programs in Nepal.

## Supplementary Information

**Additional file 1.**

## Data Availability

The data collected as part of this study are available from the corresponding author on reasonable request.

## Abbreviations

COREQ: Consolidated Criteria for Reporting Qualitative Research
CVD: Cardiovascular diseases
LTPA: Leisure-time physical activity
PA: Physical activity
STEPS: Stepwise Approach to Surveillance
T2DM: Type 2 Diabetes mellitus
