# Supplementary material for: Socio-ecological influences of leisure-time physical activity among Nepalese adults: a qualitative study
Source: BMC Public Health. 2021 Jul 22;21:1443. doi: 10.1186/s12889-021-11484-3 (PMC8296660; doi:10.1186/s12889-021-11484-3)
Supplement: Supplementary file 1 — Additional file 1. [file 12889_2021_11484_MOESM1_ESM.docx]

| **Theme** | **Question type** | **Questions** |
| --- | --- | --- |
|  | Opening | Introduction of the researchers  Explain the purpose and methodology of the study  Participants introduction:  Could you please tell me your name, age, education, occupation and where are you from? |
|  | Introduction | How do you spend a typical day?  **Probe**  What are the activities you generally perform on a normal day? |
| **Total PA** | Transition | What do you understand by physical activity?  In the last two weeks, have you engaged in any form of physical activity?  What are the activities that you typically engage in?  In your opinion, how much time should we spend on these activities in a normal day?  How do you feel about being physically active? |
| **LTPA** | Transition | How do you spend your leisure time?  What can be some of the leisure time physical activities people of your age might engage in?  How do you feel about engaging in these types of leisure time physical activities? |
|  | Key questions:  Benefits | Please tell me a few reasons why adults might engage in leisure time physical activity?  **Probes**  What are the reasons you might want to be physically active during leisure?  **Prompts**  health benefits (physical and mental health), physical appearance/maintain weight |
|  | Barriers | Please tell me a few reasons why adults might not engage in leisure time physical activity?  **Probes**  What could act as potential barriers for engaging in physical activity?  **Prompts**  Individual, Interpersonal, organisational, community factors |
|  | Facilitators | What factors could assist you in being physically active during leisure?  **Prompts**  Individual, Interpersonal, organisational, community factors |
| **For diseased group only (before closing)**  How has your participation in PA during leisure changed after being diagnosed with disease?  **Probes**  Any changes?  What assisted you to change?  What are the types of activities you now engage in?  How do you feel about being physically active? | | |
|  | Closing | Overall, In your opinion, what can be done to encourage people of your age to engage in physical activity during leisure time? |
|  |  | Do you have any remarks, suggestions or additions? |
|  |  |  |


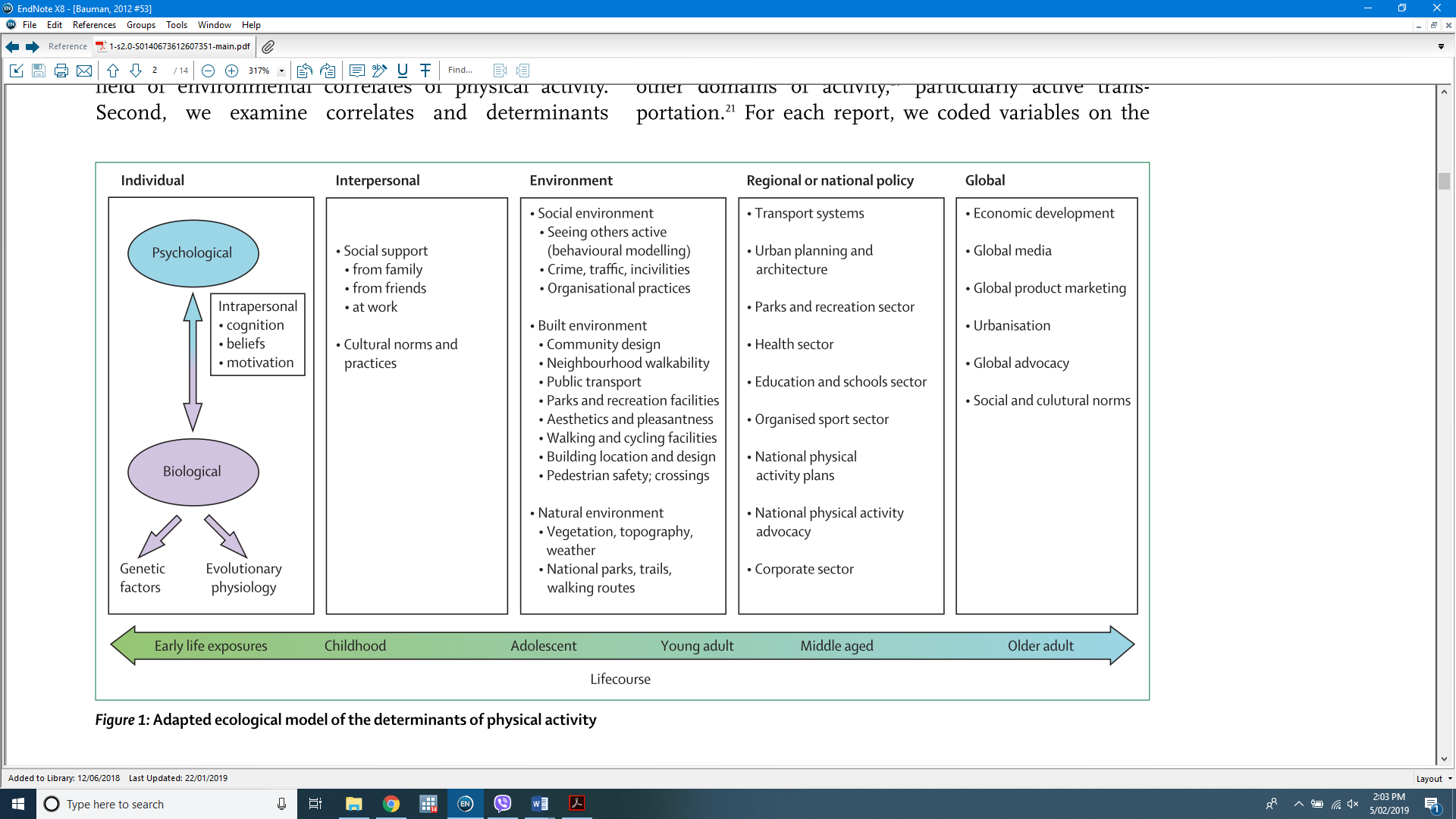


Bauman, A. E., et al. (2012). "Correlates of physical activity: why are some people physically active and others not?" The lancet **380**(9838): 258-271.
